# NeuroHeal Treatment Alleviates Neuropathic Pain and Enhances Sensory Axon Regeneration

**DOI:** 10.3390/cells9040808

**Published:** 2020-03-27

**Authors:** David Romeo-Guitart, Caty Casas

**Affiliations:** 1 Institut de Neurociències (INc) and Department of Cell Biology, Physiology and Immunology, Universitat Autònoma de Barcelona (UAB), 08193 Bellaterra, Barcelona, Spain; Caty.Casas@uab.cat; 2 Institut Necker Enfants-Malades (INEM), INSERM U1151, Laboratory “Hormonal regulation of brain development and functions”—Team 8, Université Paris Descartes, Sorbonne Paris Cité, 75015 Paris, France

**Keywords:** peripheral nerve injury, neuropathic pain, nerve regeneration, autophagy, NeuroHeal

## Abstract

Peripheral nerve injury (PNI) leads to the loss of motor, sensory, and autonomic functions, and often triggers neuropathic pain. During the last years, many efforts have focused on finding new therapies to increase axonal regeneration or to alleviate painful conditions. Still only a few of them have targeted both phenomena. Incipient or aberrant sensory axon regeneration is related to abnormal unpleasant sensations, such as hyperalgesia or allodynia. We recently have discovered NeuroHeal, a combination of two repurposed drugs; Acamprosate and Ribavirin. NeuroHeal is a neuroprotective agent that also enhances motor axon regeneration after PNI. In this work, we investigated its effect on sensory fiber regeneration and PNI-induced painful sensations in a rat model of spare nerve injury and nerve crush. The follow up of the animals showed that NeuroHeal treatment reduced the signs of neuropathic pain in both models. Besides, the treatment favored sensory axon regeneration, as observed in dorsal root ganglion explants. Mechanistically, the effects observed in vivo may improve the resolution of cell-protective autophagy. Additionally, NeuroHeal treatment modulated the P2X4-BDNF-KCC2 axis, which is an essential driver of neuropathic pain. These data open a new therapeutic avenue based on autophagic modulation to foster endogenous regenerative mechanisms and reduce the appearance of neuropathic pain in PNI.

## 1. Introduction

Peripheral nerve injury (PNI) due to complete or partial transection is a common injury that affects 13–23 people per 100,000 per year in the USA, leading to huge economic costs in healthcare [1,2]. Reconnection between the central nervous system and target organs is mandatory to ensure correct function after nerve disruption. In this way, a fast and functional regrowth of sensory axons after peripheral nerve injury (PNI) is essential to achieve fine proprioceptive and tactile sensations needed to perform many activities of normal daily life. Several works have been reported on the systemic administration of drugs, such as FK506, geldanamycin, and *N*-acetyl-cysteine, that increase the growth of sensory fibers in vitro and in vivo experimental models [3]. However, most of those compounds did not target the secondary development of hyperalgesia after PNI.

Neuropathic pain is defined as a disturbance of the somatosensory system triggered by neural injuries, which may affect up to 10% of the general population [4]. It is characterized by the appearance of spontaneous pain, dysesthesia, hyperalgesia, and allodynia. Neuropathic pain after PNI is characterized by changes in dorsal root ganglia (DRG) and dorsal horn neurons excitability, which are followed by the disturbance of the normal function of other central nervous areas such as thalamic relay nuclei, somatosensory cortex, amygdala, and others [4,5,6]. Nonetheless, despite the efforts aiming to discern its origins, the molecular mechanisms that drive neuropathic pain are multiple, making it difficult to achieve effective treatments [7,8,9,10]. In many cases, the maintenance of painful sensation is fostered by a chronic inflammation state within the dorsal horn driven by microglia-derived pro-inflammatory mediators, persistence of ionic channels imbalance in sensory neurons, and reduction of inhibitory mechanisms at the spinal cord dorsal horn [11,12]. All these molecular events lead to central sensitization, promoting excessive signaling of stimuli as painful sensations.

Several therapies such as physical exercise or electrical stimulation are proposed to reduce painful sensations [13,14], but at clinical level only pregabalin or antidepressants are used, although they have limited effects on this type of pain relief and may produce side-effects [15]. In this study, we evaluated the therapeutic potential of NeuroHeal, a combination of two repurposed drugs (Acamprosate plus Ribavirin) previously designed using artificial intelligence. We recently described the neuroprotective effect of NeuroHeal on motoneuron survival after PNI [16,17]. However, its effects on sensory reinnervation and the apparition of hyperalgesia after PNI remain unexplored. Herein, we aim to investigate this possibility using two different rat models of PNI and provide insight into the mechanisms involved.

## 2. Materials and Methods

### 2.1. In Vitro Cultures and Axonal Growth Analysis

We prepared collagen solution at 3 mg/mL by mixing rat tail collagen type I solution (Corning, Wiesbaden, Germany) with PBS (Sigma-Aldrich, Saint Louis, MO, USA), sodium bicarbonate at 0.3 mg/mL, and 10× basal Eagle’s medium (Gibco, Grand Island, NY, USA) as previously described [18]. We deposited 30 μL drops of this collagen solution in 24-well Petri dishes pretreated with poly-d-Lysine (Sigma-Aldrich) and kept them in the incubator 1 h at 37 °C and 5% CO_2_ to induce collagen gel formation. We obtained the lumbar DRG from 7-day old Sprague-Dawley rats, placed them in 30% glucose cold Gey’s balanced salt solution (Sigma-Aldrich), cleaned off meninges and nerve roots, and placed onto collagen droplets. After 30 min in the incubator, we covered the slices with a further 30 μL drop of the same collagen solution as above and after 30 min at 37 °C for collagen polymerization, we added culture medium Neurobasal (Life Technologies, Grand Island, NY, USA) supplemented with B27 (Life technologies), glutamine, and penicillin/streptomycin (Sigma-Aldrich). 

One day after culture, we renewed the culture medium containing the different treatments: H_2_O as vehicle, NeuroHeal, Acamprosate, and Ribavirin. After 2 days treatment, we fixed the DRG with cold 4% PFA solution for 1 h, washed them with TBS several times, and incubated for 48 h with primary antibodies at 4 °C. For neurite growth analysis the primary antibody was anti-mouse RT97 (1:200; Hybridoma Bank, Iowa, Iowa, USA). After washes with 0.1% Tween in TBS, we incubated the samples with donkey conjugated Alexa 594 anti-mouse (1:200; Life Technologies) overnight at 4 °C, counterstained with DAPI, washed them, and mounted with DPX (Sigma-Aldrich). 

We took sequential microphotographs with a fluorescence microscope Olympus BX51 (Olympus, Hamburg, Germany) attached to a DP73 camera and merged them with Adobe Photoshop CS6 to obtain whole DRG slice body with their neurites. To analyze neurite growth and length, whole culture images were analyzed with the help of the Neurite-J plug-in for ImageJ software. This method has been previously validated, and provides the same results as the classical Sholl analysis or manual measurements, giving a robust analysis of neurite ejections [18]. The number of neurites for each intersection from the explant was calculated and compared between sets of cultures. 

### 2.2. Drugs

For in vitro experiments, we purchased Acamprosate and Ribavirin (Sigma-Aldrich, Saint Louis, MO, USA), diluted them in H_2_O and added at 55 and 1 µM, respectively, to the DRG culture medium. In vivo treatments consisted of Acamprosate (Merck, Darmstadt, Germany) and Ribavirin (Normon, Madrid, Spain) pills ground into fine powder and dissolved in sterile saline. For 1× dose of NeuroHeal, we intraperitoneally injected 40 mg/kg of Acamprosate and 26.6 mg/kg of Ribavirin daily. In different groups of rats, doses of NeuroHeal corresponding to 0.05×, 0.2×, 1×, 1.7×, and 3× were used to assess dose-effect relationship and potential toxicity.

### 2.3. Animals and Surgery

All procedures involving animals were approved by the Ethics Committee of Universitat Autònoma de Barcelona and followed the European Community Council Directive 2010/63/EU. Female and male Sprague-Dawley rats were purchased from Harlan Laboratories, and weighed 220–250 and 250–300 g, respectively. We performed surgical procedures under anesthesia by intraperitoneal injection of ketamine (90 mg/kg, i.m.) and xylazine (10 mg/kg, i.m.). For crush nerve injury, we exposed the right sciatic nerve and crushed the nerve with a fine forceps (Dumont no. 5) in three different orientations for 30 s. Spared nerve injury (SNI) injury was performed as described previously [19]. Briefly, the right sciatic nerve was exposed at trifurcation level, the three terminal branches were gently separated proximal to the popliteal fossa, and the tibial and peroneal nerves were tightly ligated with a 6-0 suture and sectioned distal to the ligature with scissors. Extreme care was taken to avoid damage of the sural nerve. The wound was sutured by planes and disinfected with povidone iodine, and the animals were allowed to recover in a warm environment.

### 2.4. Assessment of Mechanical Allodynia

One week before surgery, all the animals were habituated to experimental devices. At different days post-injury (dpi), we evaluated the sensibility to a normal non-noxious mechanical stimulus by means of an electronic Von Frey algesimeter (Bioseb, Vitrolles, France). Briefly, we placed rats in a plastic chamber on a wire mesh floor and applied mechanical stimuli on the lateral zone of the hind paw plantar surface. We chose the lateral side because is the sensory territory of the sural nerve [20]. A 0.4 mm non-noxious pointed probe was gently applied to the test site, slowly increasing the pressure until the animal withdrew the paw in response to the stimulus, considering the force (in grams) as withdrawal threshold. Three different measurements per test site with 5 min-interval between each one were applied and the mean was calculated. All the experiments to study nociceptive response were performed by a researcher blinded to the treatment received by each rat group.

### 2.5. Electrophysiological Test

To determine motor axon regeneration, we performed electrophysiological tests at different dpi. Rats were anesthetized with ketamine/xylazine (100:10 mg/kg weight, i.p), and the sciatic nerve was stimulated by transcutaneous electrodes placed at the sciatic notch by single pulses (20 µs). After the stimulation, we analyzed the presence of compound muscle action potential (CMAP) by placing electrodes on gastrocnemius and plantar interosseous muscles. The evoked action potentials were displayed on a storage oscilloscope (Synergy Medelec, Viasys HealthCare, Conshohocken, PA, USA) at settings appropriate to measure the amplitude (mV) and latency (ms) to the onset after every stimulus. After testing, animals were allowed to recover in a warm environment. 

### 2.6. Tissue Processing for Histology

We euthanized at 35 dpi the animals with pentobarbital (60 mg/kg i.p), and intracardially perfused them with saline solution containing heparine (10 U/mL) followed by infusion of 4% paraformaldehyde in 0.1M PBS buffer. We collected the L4-L5 spinal cord segments, which were post-fixed during 2 h with the same fixative solution and then placed in a 30% sucrose solution for cryopreservation at 4 °C. The spinal cord samples were embedded in Tissue-tek (Sakura Finetek, Alphen aan den Rijn, The Netherlands) and cut into serial slices of 20 µm thickness (three series of 10 slices with 10 slides each one) with a cryotome (Leica, Heidelberg, Germany) and kept them at −20 °C until used.

### 2.7. Immunohistochemistry and Image Analysis

Spinal cord slices were washed with Tris-buffered saline (TBS), treated with TBS-Glycine 0.1 mM and with blocking solution TBS-0.3% Triton-X-100 with 10% donkey serum for 1 h at RT. Then, we incubated the slices overnight at 4 °C with the primary antibody rabbit anti-ionized calcium binding adaptor molecule 1 (Iba1; 1:1000, Wako). After several washes with TBS-Tween-20 at 0.1%, we added specific donkey-Cy3 against primary antibody (1:200; Jackson Immunoresearch, Ely, United Kingdom) for 1 h and 20 min at RT. Besides, the exceeding secondary antibody was removed washing with TBS-0.3%-Triton-X-100 and the slices were counterstained with fluorescent green NeuroTracer Nissl Stain (Molecular Probes, Leiden, Netherlands) and DAPI (Sigma, St Louis, MO, USA). The slices were washed with TBS, TB, and were mounted with Fluoromount-G mounting medium (SouthernBiotech, Birmingham, AL, USA). Tissue sections to be compared were processed in parallel the same day.

We analyzed Iba1 labeling to assess microgliosis. We acquired images from at least five spinal cord sections (separated by 200 μm between pairs) from L4-L5 spinal segments immunolabeled against Iba1 at 20× with the aid of a digital camera (Olympus DP76, Olympus, Hamburg, Germany) attached to a microscope (Olympus BX51, Olympus, Hamburg, Germany). The integrated density of a ROI covering the dorsal horn was calculated. 

### 2.8. Western Blot

At 7 dpi, another set of animals was euthanized to obtain L4-L5 dorsal horn from control and SNI animals. Samples were snap frozen in liquid nitrogen for storage and were processed by homogenization in lysis buffer (20 mM HEPES, pH 7.2, 250 mM sucrose, 1 mM EDTA, 1 mM EGTA) and a cocktail of protease (Sigma-Aldrich) and phosphatase inhibitors (Roche, Darmstadt, Germany) in a Pellet pestle (Sigma-Aldrich) on ice to obtain the cytosolic fraction. We centrifuged the homogenate at 800× *g* for 20 min at 4 °C, collected the supernatant, and quantified the protein content by BCA assay (Pierce Chemical Co., Dallas, TX, USA). Equal amounts of cytosolic protein fractions from L4-L5 segments of each animal were loaded onto 10–15% SDS-polyacrylamide gels to perform electrophoretic separation of the proteins. Then, we transferred the proteins to a PVDF membrane in a BioRad cuvette system in 25 mM Tris, pH 8.4, 192 mM glycine, 20% (*v*/*v*) methanol. Membranes were blocked with 6% milk in tris-buffered saline (TBS) plus 0.1% Tween-20 for 1 h at room temperature and then incubated at 4 °C overnight with the following primary antibodies: mouse anti-β-actin (1:5000;Sigma-Aldrich), mouse anti-GAPDH (1:5000; Millipore), mouse anti-p62 (1:500; BD Transduction Laboratories), mouse anti-ATG5 (1:1000;Nanotools), rabbit anti-LC3 (1:500; Proteintech), rabbit anti-pULK1 (1:1000; Proteintech), goat anti-P2X4 (1:500; Sigma-Aldrich), rabbit anti-KCC2 (1:1000;Millipore), sheep anti-BDNF (1:500; Millipore) and rabbit anti-Iba1 (1:500; Wako). After several washes, the membranes were incubated for 1 h with an appropriate secondary antibody conjugated with horseradish peroxidase (1:5000, Vector). The membrane was visualized using a chemoluminescent method (ECL Clarity Kit, Bio-Rad Laboratories, Hercules, CA, USA), and the images were captured and quantified with Image Lab Software (Bio-Rad Laboratories).

### 2.9. Statistical Analysis

All values are presented as mean ± standard error of the mean (SEM). Statistical analyses were performed using GraphPad Prism 7 software by one or two-way analysis of variance (ANOVA) followed by Bonferroni’s multiple comparison tests or by unpaired *t*-tests (one or two sided). We analyzed the in vitro sensory growth and the Von Frey filament test values in vivo by two-way ANOVA followed by Bonferroni’s multiple comparison. We performed statistical analysis (ANOVA, two-way) to determine if the variant “sex” has an effect on the vehicle or NeuroHeal-treated animals. Since we did not observe any significant differences, we decided to pool both sexes in the final statistical study. For Western blotting and immunohistochemistry analysis, we performed one-way ANOVA followed by Bonferroni’s multiple comparison. A *p*-value of 0.05 was taken to indicate significant difference between groups.

## 3. Results

### 3.1. NeuroHeal Enhances Regeneration of Sensory and Motor Axons

**Figure 1:**
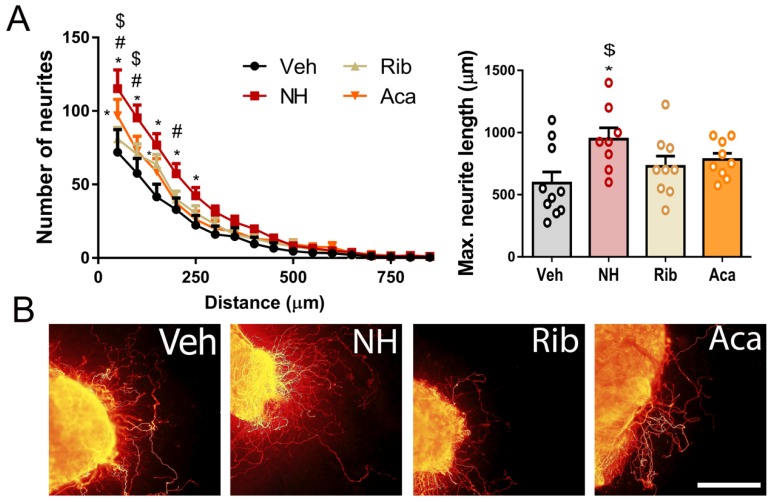
NeuroHeal increases neuritogenesis in vitro. (**A**) Graphs showing the number of neurites per intersection (left) and the maximum neurite length (right) sprouted out from collagen-embedded dorsal root ganglia (DRG) explants (*n* = 8–10 biological replicates, ANOVA, post hoc Bonferroni, * *p* < 0.05 vs. Veh, # vs. Aca and $ vs. Rib, combined with *t*-test). (**B**) Representative microphotographs of collagen embedded DRGs treated with Vehicle (Veh), NeuroHeal (NH), Acamprosate (Aca), and Ribavirin (Rib). Scale bar = 250 µM.

To test whether NeuroHeal increases the regeneration of sensory axons, as it does for motor ones [17,21,22], we performed in vitro and in vivo experiments. We treated collagen-embedded DRG explants with NeuroHeal, Acamprosate or Ribavirin, and analyzed the number of neurites sprouting out from the explants and neurite length. NeuroHeal-treated DRGs presented a significant increase in the number and maximum length of neurites outgrowth compared to vehicle-treated ones (Figure 1). We observed that NeuroHeal increased significantly the number of neurites respect to the vehicle group whereas Acamprosate or Ribavirin alone did not (Figure 1). Indeed, NeuroHeal-treated DRG presented significant differences in the number of neurites compared with Acamprosate and Ribavirin. Lastly, NeuroHeal significantly increased the maximum neurite length compared to Ribavirin. Altogether, these results suggested that only the synergic activity of both drugs together, conforming that NeuroHeal can enhance the neuritogenesis in DRGs.

**Figure 2:**
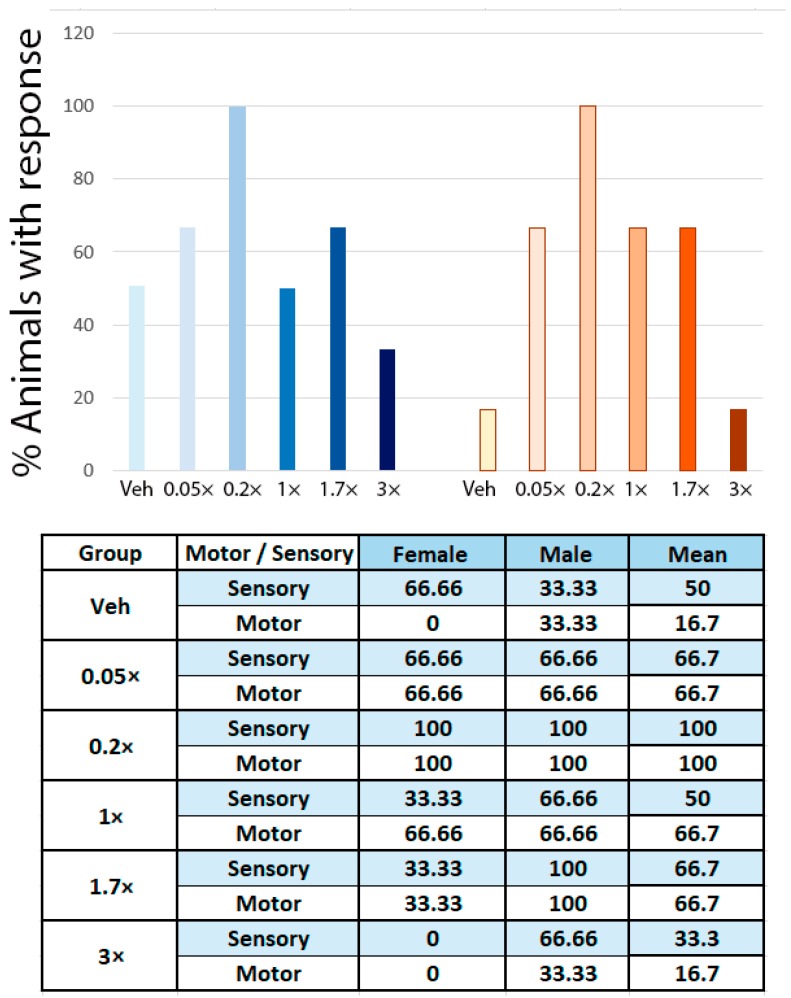
Graph and table showing the percentage of animals with sensory (Von Frey test) or motor reinnervation (compound muscle action potential (CMAP) response at plantar interossei) at 25 days post crush in different experimental groups.

Next, we tested NeuroHeal effects in vivo in a rat model of sciatic nerve crush. Different rat groups were treated with vehicle or different doses of NeuroHeal (0.05×, 0.2×, 1×, 1.7× or 3×), and they were assessed by mechanical algesimetry tests with an electronic Von Frey device at 15, 25, and 35-days post injury (dpi) (Table S1 for 25 and 35 dpi). A total of 100% of 0.2× animals presented sensory response at 25 dpi, while only 50% of vehicles had it, indicating an increased sensory axon regeneration (Figure 2). We also evaluated motor axon regeneration by electrophysiological results, observing that 100% of the animals from 0.2× dose had a response at 25 dpi, compared to 33% of the vehicle group (Figure 2). In the same direction, we observed that NeuroHeal-treated animals had a significant increase in the CMAP amplitude at gastrocnemius and plantar interossei muscles at 35 dpi (Table 1), which indicates an enhanced motor axon regeneration. Animals that received 0.05×, 0.2×, 1×, and 1.7× dose of NeuroHeal presented a significant increase, while 3× did not. Altogether, this data suggests a dose-response effect of NeuroHeal to improve sensory and motor axon regeneration after PNI. Additionally, we evaluated the weight evolution of NeuroHeal-treated animals, and we found no significant differences between groups although animals that received high doses of NeuroHeal had slight reduction of weight gain (Figure S1).

### 3.2. NeuroHeal Reduces Hyperalgesia after Peripheral Nerve Injury

**Figure 3:**
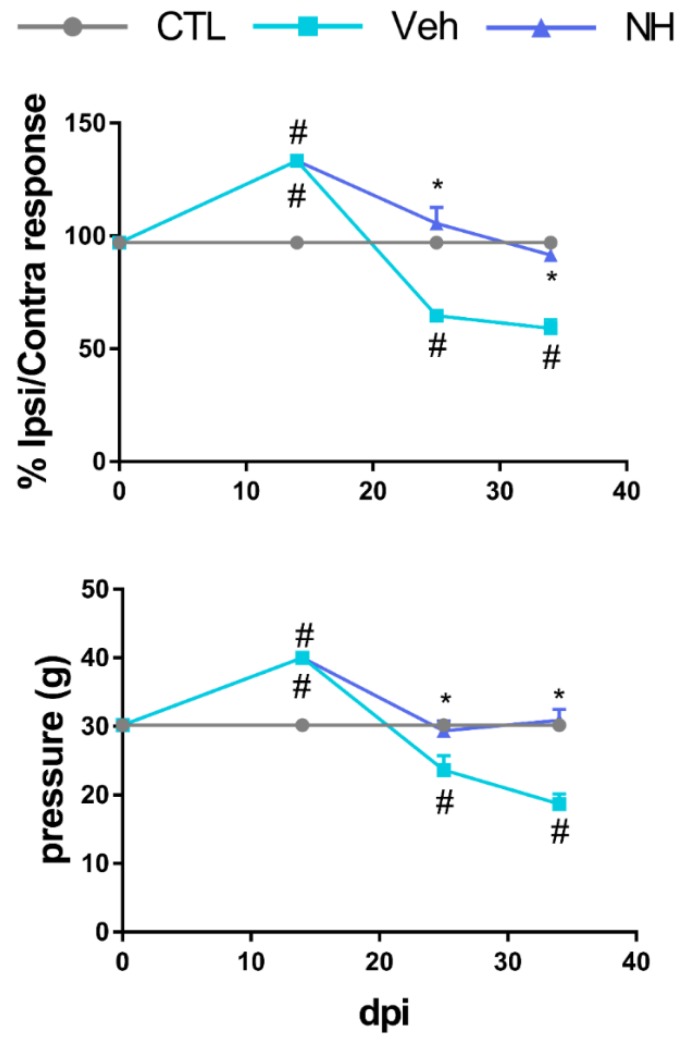
NeuroHeal reduces hyperalgesia after peripheral nerve injury (PNI). Changes in mechanical sensory thresholds recorded at the lateral side of the right hindlimb from Control, Vehicle (Veh), or 0.2× NeuroHeal (NH) dose at different days post-injury (dpi) post crush. (*n* = 5 for CTL, 6 for other groups, ANOVA, post hoc Bonferroni, * *p* < 0.05 vs. Veh, # *p* < 0.05 vs. CTL).

To determine the effects of NeuroHeal in neuropathic pain, we followed up the sciatic-crushed animals by performing Von Frey test at different time-points after injury [19]. This animal model allows axon regrowth and triggers neuropathic pain, mimicking clinic situations in patients with PNI. Among all the NeuroHeal-treated groups tested (Table S1), animals that received 0.2× daily dose of NeuroHeal presented a similar nociceptive stimulation threshold as control animals at 25 dpi, while vehicle-treated ones presented a significant reduction of it (Figure 3, Figure S2 for males and females, and Table S1). NeuroHeal effects appeared at 25 dpi, when the first sensory axons reinnervate the footpaths, and were maintained until 35 dpi, indicating that it has a stable effect of blocking nociception (Figure 3). These data suggested that NeuroHeal treatment may reduce the presence of neuropathic pain after PNI. 

### 3.3. NeuroHeal Reduces Mechanical Allodynia

We assessed the effect of NeuroHeal in the spared nerve injury (SNI) model, which is widely used to study the apparition of neuropathic pain. This animal model is characterized by a marked neuropathic pain apparition during the first days after the injury. Moreover, if the injury is maintained and axons cannot re-grow within the nerve, the painful sensations are sustained over time.

**Figure 4:**
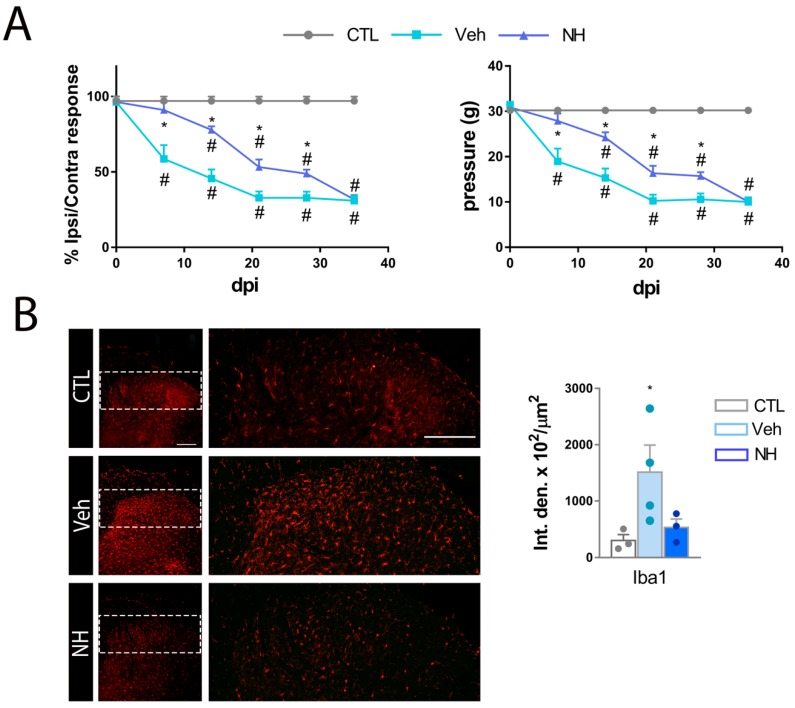
NeuroHeal reduces mechanical allodynia after SNI. (**A**) Changes in mechanical sensory thresholds recorded at the ipsilateral side of the right hindlimb from Control, Vehicle (Veh), or 0.2× NeuroHeal (NH) dose at different dpi post SNI (*n* = 5 CTL, 8 for other groups, ANOVA, post hoc Bonferroni, * *p* < 0.05 vs. Veh, # *p* < 0.05 vs. CTL). (**B**) Top, representative fluorescence microphotographs of the ipsilateral dorsal horns of the spinal cord from CTL or SNI injured animals immunolabeled against Iba1. Bottom, bar graph of the average of Iba1 immunoreactivity intensities within dorsal horn for different experimental groups at 35 dpi (*n* = 3–4 biological replicates, ANOVA, post hoc Bonferroni, * *p* < 0.05 vs. CTL, Scale bar = 200 μm).

All the groups presented similar Von Frey thresholds in the hind paw before surgery. After SNI, mechanical thresholds in the paw contralateral to surgery were similar to the baseline values in all groups (Table 2). Both groups of animals progressively developed mechanical allodynia in the injured limb, manifested as a significant reduction in the mechanical threshold in the paw ipsilateral to the injury. The vehicle group presented a significant reduction of mechanical threshold starting from 7 dpi. In contrast, the animals treated with NeuroHeal did not develop mechanical hypersensitivity until 14 dpi (Figure 4A and Figure S3). By 21 dpi, animals receiving NeuroHeal presented less mechanical hypersensitivity than those receiving vehicle, suggesting that the treatment promoted an attenuated neuropathic allodynia. Hence, we wonder whether NeuroHeal may have had long-lasting analgesic effect. For this reason, we removed NeuroHeal treatment at 21 dpi and extended the follow up by two extra weeks. Animals from the vehicle group reached a plateau of mechanical threshold in their response. The animals that previously received NeuroHeal sustained the previous threshold one week later although the values dropped to reach the same as the vehicle group by 35 dpi (Figure 4A). To confirm that NeuroHeal antinociceptive effects were not promoted by general anesthesia, we compared contralateral values from Veh- and NeuroHeal-treated animals (Table 2). We did not observe significant differences between the left hindlimb of both groups, confirming that NeuroHeal reduces nociceptive threshold after SNI. 

Lastly, we analyzed microglial reactivity by Iba1 labelling at two weeks after NeuroHeal removal (35 dpi) in the dorsal horn, observing that NeuroHeal significantly reduced microgliosis compared to vehicle group (Figure 4B). Therefore, NeuroHeal has long-lasting anti-inflammatory effects for at least two weeks.

### 3.4. Autophagy Impairment is Resolved by NeuroHeal Treatment

**Figure 5:**
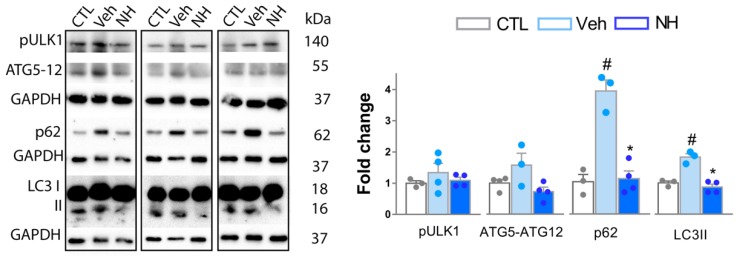
NeuroHeal treatment resolves autophagic blockage triggered after SNI. Western blot and corresponding bar graphs of the quantification of different proteins related to autophagy (pULK (Ser-555), ATG5, LC3-II, and p62) in the dorsal horn from L4-L5 spinal cords from Control (CTL), Vehicle (Veh), or NeuroHeal (NH) treated animals. (*n* = 3–4 biological replicates, ANOVA, post hoc Bonferroni, * *p* < 0.05 vs. Veh, # *p* < 0.05 vs. CTL).

Different studies suggested an essential role of macro-autophagy, hereinafter referred to as autophagy, in nociception [23,24,25], and we recently observed that NeuroHeal induces autophagy in certain conditions [26]. In cells undergoing autophagy, phagophore formation initiates after the Unc-51 like autophagy activating kinase 1 (ULK1) activation, and its elongation is regulated by two ubiquitin-like reactions: the first leading to the formation of the complex ATG12-ATG5-ATG16L1; and the second, involves the conjugation of the microtubule-associated protein light chain 3 (MAP-LC3/ATG8/LC3) to phosphatidylethanolamine at the autophagosome membrane to form autophagosome-associated LC3-II. Once the autophagosome is created, it acquires the ability to bind autophagic substrates and/or proteins that mediate cargo selectivity (including sequestosome 1 (p62/SQSTM1)). Seeking to analyze the state of autophagy after NeuroHeal treatment, we determined by Western blot the levels of phosphorylated Ulk-1 at Ser555 residue (pUlk1), as an autophagy initiator, and levels of LC3II, ATG5-ATG12, and p62, to monitor autophagic flux in the dorsal horn of the L4-L5 segment [27]. We observed that pUlk1 levels did not significantly change in all groups with respect to control animals (Figure 5). Accordingly, no changes were observed in the levels of ATG5-ATG12 conjugate. In contrast, we observed a significant increase in the levels of LC3-II and p62 in vehicle group compared to control, while the NeuroHeal group presented normalized levels (Figure 5). When these markers are increased, they are indicative that normal autophagy flux is blocked. Altogether, these results suggested that, at this time point, there was no evidence of autophagy initiation dissimilar to baseline control, but signs of autophagy flux blockage promoted by injury. Additionally, NeuroHeal treatment seemed to revert this problem. 

### 3.5. NeuroHeal Increases KCC2 Levels in Dorsal Spinal Horn

**Figure 6:**
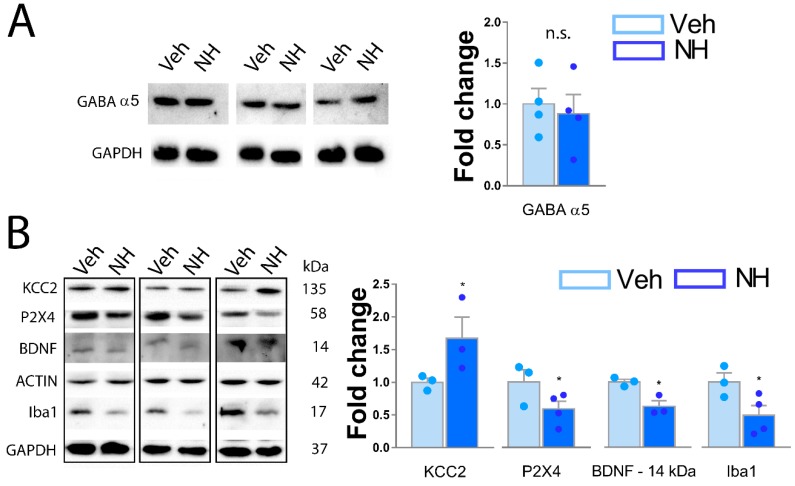
Modulation of P2X4-BDNF-KCC2-Iba1 axis by NeuroHeal. (**A**) Western blot and corresponding bar graph of the quantification α5 GABA subunit in the dorsal horn from L4-L5 spinal cords from Vehicle (NH) or NeuroHeal (NH) treated SNI animals. (*n* = 4 biological replicates, *t*-test, * *p* < 0.05 vs. Veh; n.s. = nonsignificant). (**B**) Western blot and corresponding bar graph of the quantification of P2X4, BDNF (14kDa), KCC2 monomer, and Iba1 subunit in the dorsal horn from L4-L5 spinal cords from Vehicle (Veh) or NeuroHeal (NH) treated SNI animals. (*n* = 3–4 biological replicates, *t*-test, post hoc Bonferroni, * *p* ≤ 0.05 vs. Veh).

Several molecular pathways have been described to drive the apparition of neuropathic pain. Seeking to understand which can be modulated by NeuroHeal, we first analyzed the inhibitory state of dorsal neurons by the abundance of GABA α-5 subunit, linked to the increase in painful sensations [28]. As shown in Figure 6A, there were no differences between Veh and NeuroHeal groups, suggesting that these receptors were not responsible for the attenuation of the neuropathic pain like behavior produced by NeuroHeal (Figure 6A). On the other hand, P2X4-dependent microglial activation increases BDNF release in dorsal horn, contributing to the maintenance of neuropathic pain by the downregulation of KCC2 transporter in dorsal neurons [29,30,31]. Hence, we analyzed by Western blot the levels of mature BDNF monomer (14 kDa), the glycosylated form of P2X4 which is the form presented in the cellular membrane [32], and the total levels of KCC2. We observed that NeuroHeal treatment significantly reduced the presence of mature BDNF and the levels of glycosylated P2X4, with respect to the vehicle group (Figure 6B). Moreover, NeuroHeal significantly increased the abundance of KCC2 within the dorsal horn of spared nerve injured animals with respect to those treated with vehicle (Figure 6B). Finally, we observed a reduction of Iba1 levels, confirming the data described in Figure 3. Altogether, these results suggest that NeuroHeal exerted an effect in microglial P2X4-BDNF axis activity, probably contributing to the observed increased levels of KCC2 linked to neuropathic pain maintenance.

## 4. Discussion

Neuropathic pain is presented in patients after PNI, spinal cord injury, or in somatosensory system disorders, affecting their daily life. Many efforts are focused on obtaining an effective therapy to modulate nociception. Here, we describe that the treatment with NeuroHeal enhances sensory axon regeneration while averts neuropathic pain apparition after PNI in two rat models. We also described that NeuroHeal mechanism of action may involve the resolution of autophagy within dorsal horn neurons, and the modulation of the P2X4-BDNF-KCC2 axis. Both events may re-establish the ionic channel imbalance that characterizes central sensitization present in neuropathic pain.

NeuroHeal was in silico designed to promote neuroprotection, enhance axonal regeneration, and reduce neuropathic pain after nerve root avulsion [17,33]. We confirmed its neuroprotective effect in different in vivo models of motoneuron death after PNI [16,17], and we also tested its ability to increase motor axonal regeneration [22]. Nonetheless, its effects in the sensory system after nerve trauma remained elusive. Herein, we shown in vivo evidence describing the positive effects of NeuroHeal enhancing sensory function recovery after PNI. 

Our first discovery is that NeuroHeal increases the regeneration of sensory axons. Although unexplored, NeuroHeal may be increasing sensory axon regeneration through the modulation of autophagy, as it does for motor axons [22]. Autophagy is a crucial cellular process in neurons/peripheral nerves after PNI because it (i) removes the degenerated axons allowing for a permissive microenvironment for regeneration [34], (ii) enhances axonal growth and functional recovery after spinal cord injury [35], and (iii) increases sensory and motor axon regeneration [22,36]. Moreover, autophagy flux disruption has been described as sufficient to block axon regeneration [37]. Different ATG proteins have been linked with the regenerative process. Ulk1 protein, which is essential for autophagy initiation [27], coordinates axonal extension of sensory axons by the endocytosis of NGF and the consequent modulation of TrkA receptor trafficking and signaling [38]. ATG5 overexpression facilitates a more pro-regenerative phenotype to spinal motoneurons after PNI [22].

Xie et al., 2017 described that the genetic ablation of the pro-regenerative protein Advilin triggers neuropathic pain, crosslinking sensory axon regeneration with nociception. The same article demonstrated that the permanent activation of the pro-regenerative machinery in sensory axons elicits neuropathic pain in mice [39]. Hence, accelerated axonal regeneration seems to be an attractive strategy to ameliorate pain. Nonetheless, several therapies based on neurotrophic factors, such as NGF, increased axon regeneration but also triggered pain. In this way, the treatment with an antibody against NGF resulted in the amelioration of different types of pain in clinical trials [40]. Therefore, our study characterizes the first drug-based therapy that enhances axon regeneration and attenuates the apparition of mechanical allodynia, making NeuroHeal a better approach than those based on NGF or other neurotrophic factors [41].

Autophagy is a cell-protective mechanism whenever it is appropriately induced and resolved. This concept is elegantly reflected in neurodegenerative diseases or motoneuron death after PNI [26,42,43]. Besides, some articles reported that autophagy flux is blocked within the dorsal horn neurons after chronic constriction injury (CCI) or SNI, which may trigger neuropathic pain [24]. Moreover, enhanced autophagy in DRG can prevent the chronicity of pain [44]. Autophagy is also involved in microgliosis. TLR4-dependent microglial activation blocks autophagic flux within spinal cord sensory neurons, which leads to pain behaviors [23]. Microglial autophagy induction reduces cytokine production by inhibiting inflammasome formation, which could lead to the reduction of neuropathic pain [25]. Lastly, we did not discard the hypothesis that NeuroHeal may be inducing autophagy in other cell types such as GABAergic interneurons or Schwann cells [45,46], which alleviate painful behaviors.

NeuroHeal-induced activation of NAD-dependent deacetylase sirtuin-1 (SIRT1) is a crucial mediator of its neuroprotective and regenerative effects [16,17]. SIRT1 modulates autophagy in different ways [47]. Although unexplored here, SIRT1 may be the primary mediator of the autophagy and analgesic effects of NeuroHeal within dorsal neurons, because its deacetylase activity reduces neuropathic pain after PNI [48,49]. In this way, a recent article described that the SIRT1-activator resveratrol increases KCC2 levels in human Rett syndrome neurons, which fits with our results [50]. Therefore, further studies are needed to confirm the role of SIRT1 in the analgesic effects of NeuroHeal and to elucidate the underlying molecular mechanisms. 

NeuroHeal exerts a long-lasting anti-microgliosis effect for at least two weeks, which suggests that its modulation of nociception may be mediated by the attenuation of the inflammatory reaction that provokes central sensitization. Taking each drug separately, Acamprosate reduces mechanical and thermal hyperalgesia. Nonetheless, these effects are mediated by anti-oxidant activities instead of an anti-inflammatory effect [51]. Indeed, Acamprosate may increase KCC2 levels by the inhibition of NMDA receptors, which blocks the degradation of KCC2 by the calcium-activated protease calpain [52]. Ribavirin may yield anti-inflammatory effects by modulating micro- and astrogliosis, as it does in experimental autoimmune encephalomyelitis rat models [53,54]. Moreover, TLR4 levels, which are related to nociception, is one of Ribavirin putative targets in other immunological responses [23,55,56,57]. Lastly, it is important to remark that 100 mg/kg of Acamprosate has no effects alleviating pain [51], and that we administered it at 8 mg/kg, which reinforces the synergistic effect of NeuroHeal towards this pathophysiological phenomenon. Therefore, both drugs are needed together to promote the dual effect of NeuroHeal: reduce neuropathic pain and enhance sensory axonal regeneration. 

Overall, the data presented here provide evidence that NeuroHeal could be an effective therapy after peripheral nerve injury, enhancing regeneration while attenuating the apparition of neuropathic pain. Our results also suggest that autophagy modulation opens novel therapeutic avenues to ameliorate life quality of patients that suffer from different types of pain, including those that originated within the nervous system.

## 5. Study Limitations and Future Directions

The first limitation of this study was that the two molecular pathways involved, KCC2-P2X4-BDNF and the autophagic flux, were assessed in a time-window. Autophagy and inflammatory responses are highly dynamic events and the analysis of their evolution during different times after PNI is important. However, due to the different experimental groups and models, we decided to focus on a specific day post-injury. Moreover, it would be interesting to analyze the effects of NeuroHeal after the pharmacological or genetic modulation of the molecules and processes involved.

This work has opened novel windows to test the therapeutic effect of NeuroHeal. Does NeuroHeal ameliorate neuropathic pain when it is already present? Does NeuroHeal modulate another type of pain? We are currently exploring other possible therapeutic effects of NeuroHeal.

## 6. Conclusions

In summary, we describe the therapeutic potential and possible mechanism of action of NeuroHeal, an agent previously discovered using machine learning and computational tools. Neuroheal attenuates the apparition of neuropathic pain after peripheral nerve injury.

## 7. Patents

NeuroHeal has a granted European patent. 

## Figures and Tables

**Table 1:** Mean amplitudes (±SEM) values of CMAP recordings obtained during follow-up post-crush from Gastrocnemius (Top), and plantar interossei muscles (Bottom) in Veh- or NeuroHeal-treated animals (NH = NeuroHeal). (*n* = 5 for CTL, 6 for other groups, ANOVA, post hoc Bonferroni, * *p* < 0.05 vs. Veh).

	**Gastrocnemius CMAP Amplitude (mV)**					
**dpi**	**Veh**	**NH 0.05×**	**NH 0.2×**	**NH 1×**	**NH 1.7×**	**NH 3×**
**15**	1.93 ± 0.4	1.02 ± 0.11	3.273 ± 0.61	0.92 ± 0.26	2.783 ± 0.83	1.04 ± 0.20
**25**	9.99 ± 1.25	10.51 ± 0.09	13.79 ± 1.08	14.22 ± 1.47	14.24 ± 0.47	10.8 ± 1.19
**35**	24.4 ± 1.59	29.94 ± 1.52 *	31.43 ± 1.46 *	28.79 ± 2.51	29.08 ± 1.25 *	25.25 ± 2.80
	**Plantar Interossei CMAP Amplitude (mV)**					
**dpi**	**Veh**	**NH 0.05×**	**NH 0.2×**	**NH 1×**	**NH 1.7×**	**NH 3×**
**15**	0	0	0	0	0	0
**25**	0.015 ± 0.015	0.132 ± 0.05	0.29 ± 0.06	0.313 ± 0.184	0.252 ± 0.109	0.02 ± 0.02
**35**	0.828 ± 0.09	1.20 ± 0.10 *	1.292 ± 0.11 *	1.335 ± 0.16 *	1.13 ± 0.08 *	0.684 ± 0.13

**Table 2:** Mechanical sensory thresholds (mean ± SEM) recorded at the lateral side of the contralateral hindlimb from males and females treated with Vehicle or NeuroHeal after spared nerve injury at different dpi.

	Pressure (g)			
	Male		Female	
dpi	Veh	NH 0.2×	Veh	NH 0.2×
7	32.8 ± 1.17	31.29 ± 1.18	33.31 ± 0.09	29.86 ± 0.64
14	31.5 ± 0.56	31.70 ± 0.73	31.96 ± 1.14	30.25 ± 0.91
21	31.25 ± 0.42	30.69 ± 0.25	30.875 ± 0.40	30.55 ± 1.33
28	32.72 ± 0.24	31.84 ± 0.71	31.74 ± 0.23	31.28 ± 1.40
35	31.88 ± 0.33	32.24 ± 0.55	32.65 ± 0.36	31.47 ± 0.50

## Supplementary Materials

The following are available online at https://www.mdpi.com/2073-4409/9/4/808/s1, Figure S1: Body weight gain is not disturbed by NeuroHeal treatment; Figure S2: Changes in mechanical sensory thresholds recorded at the lateral side of the right hindlimb from Control, Vehicle (Veh), or 0.2× NeuroHeal (NH) dose at different dpi post crush for males and females. (*n* = 2–3 for CTL, 3 for other groups, ANOVA, post hoc Bonferroni, * *p* < 0.05 vs. Veh, # *p* < 0.05 vs. CTL); Figure S3: Changes in mechanical sensory thresholds recorded at the lateral side of the right hindlimb from Control, Vehicle (Veh), or 0.2× NeuroHeal (NH) dose at different dpi post SNI for males and females. (*n* = 2–3 CTL, 4 for other groups, ANOVA, post hoc Bonferroni, * *p* < 0.05 vs. Veh, # *p* < 0.05 vs. CTL); Table S1: Mechanical sensory thresholds (mean ± SEM) recorded at the lateral side of the ipsilateral hindlimb from Vehicle and treated animals with different doses of NeuroHeal at 25 and 35 days post crush (NH = NeuroHeal). (*n* = 3 per sex, 6 in total, ANOVA, post hoc Bonferroni, * *p* < 0.05 vs. Veh).
