# Supplementary material for: NeuroHeal Treatment Alleviates Neuropathic Pain and Enhances Sensory Axon Regeneration
Source: Cells. 2020 Mar 27;9(4):808. doi: 10.3390/cells9040808 (PMC7226810; doi:10.3390/cells9040808)
Supplement: Supplementary file 1 [file cells-09-00808-s001.pdf]

Supplementary figures:

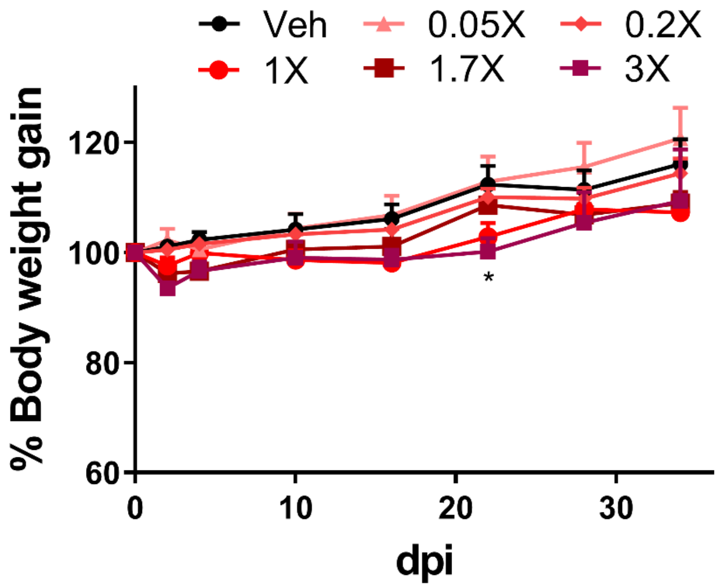

Figure S1. Body weight gain is not disturbed by NeuroHeal treatment. Graph showing the mean values (± SEM) of body weight evolution for Vehicle (Veh) or different NeuroHeal-treated (NH) groups compared with the previous day of the injury until 35 dpi ($n = 6$, ANOVA, post hoc Bonferroni, $*p < 0.05$ vs. Veh).

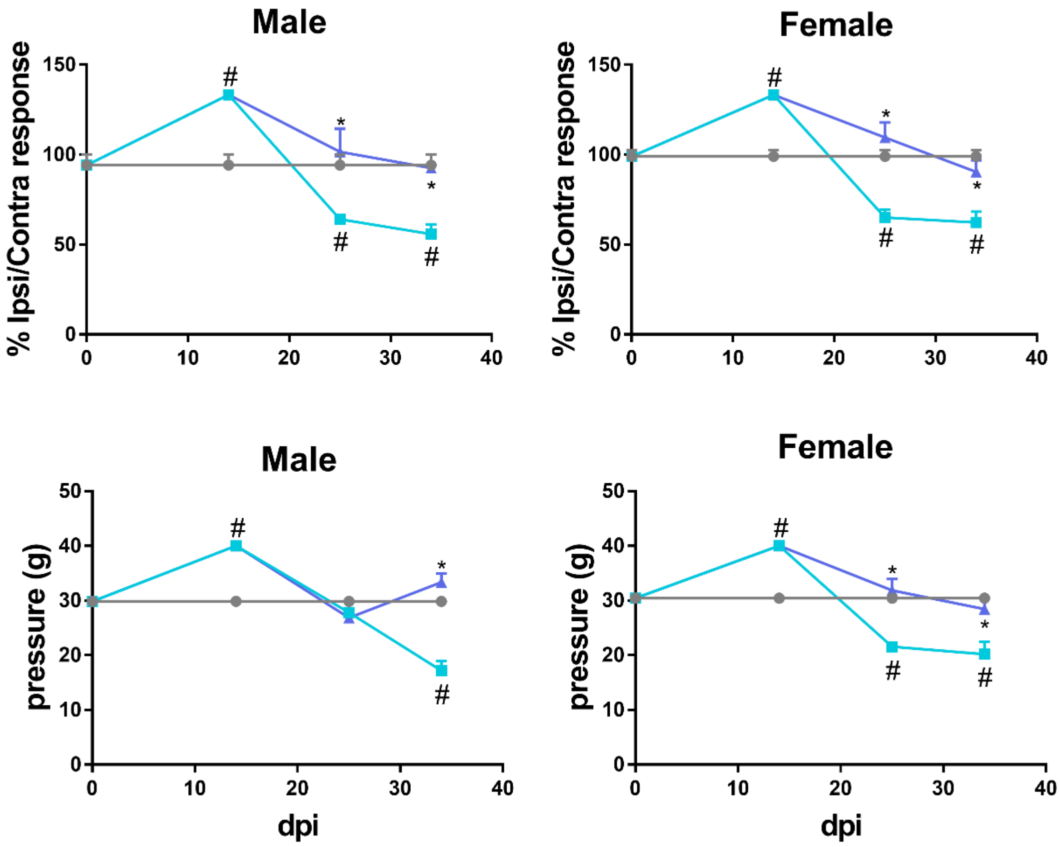

Figure S2: Changes in mechanical sensory thresholds recorded at the lateral side of the right hindlimb from Control, Vehicle (Veh), or 0.2× NeuroHeal (NH) dose at different dpi post crush for males and females. (n = 2–3 for CTL, 3 for other groups, ANOVA, post hoc Bonferroni, *$p < 0.05$ vs. Veh, # $p < 0.05$ vs. CTL).

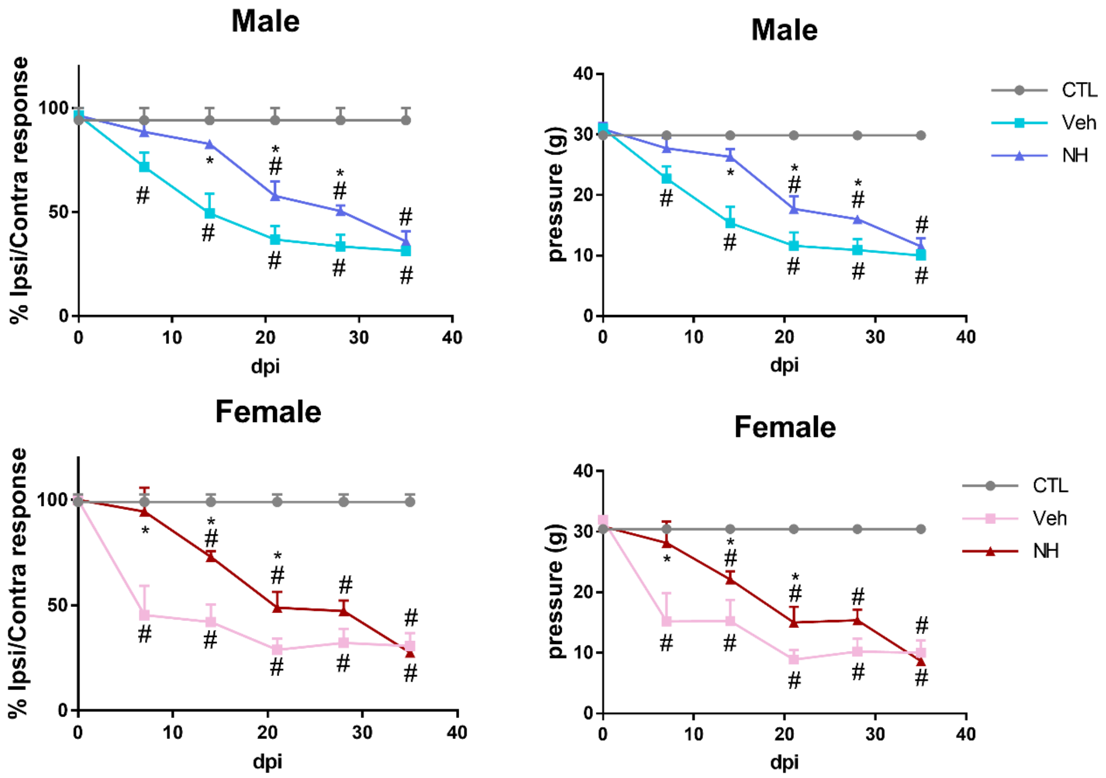

Figure S3. Changes in mechanical sensory thresholds recorded at the lateral side of the right hindlimb from Control, Vehicle (Veh), or 0.2× NeuroHeal (NH) dose at different dpi post SNI for males and females. (n = 2–3 CTL, 4 for other groups, ANOVA, post hoc Bonferroni, *$p < 0.05$ vs. Veh, # $p < 0.05$ vs. CTL).

Supplementary table:

Table S1. Mechanical sensory thresholds (mean ± SEM) recorded at the lateral side of the ipsilateral hindlimb from Vehicle and treated animals with different doses of NeuroHeal at 25 and 35 days post crush (NH = NeuroHeal). (n = 3 per sex, 6 in total, ANOVA, post hoc Bonferroni, * < p 0.05 vs. Veh).

**Pressure (g) at 25 dpi**						
**Group**	**Veh**	**NH 0.05×**	**NH 0.2×**	**NH 1×**	**NH 1.7×**	**NH 3×**
**Male**	27.8 ± 0	26.2 ± 3.1	26.9 ± 0.2	28.7 ± 1.5	30.4 ± 2.4	0 animals with response
**Female**	21.6 ± 0.3	28.2 ± 2.9	31.9 ± 1.7 *	33.4 ± 0 *	33.6 ± 0.1 *	24.9 ± 1.0
**Both sexes**	23. 6 ± 1.7	27.2 ± 2.2 *	29.4 ± 1.3 *	30.3 ± 1.6 *	31.7 ± 1.6 *	24.9 ± 1.0

**Pressure (g) at 35 dpi**						
**Group**	**Veh**	**NH 0.05×**	**NH 0.2×**	**NH 1×**	**NH 1.7×**	**NH 3×**
**Male**	17.2 ± 1.4	29.3 ± 1.5 *	33.3 ± 1.3 *	22.6	30.5 ± 0.5 *	25.3 ± 0.4
**Female**	20.2 ± 1.9	29.3 ± 1.3 *	28.4 ± 1.7 *	27.9 ± 1.4 *	24.7 ± 2.9	18.2 ± 2.8
**Both sexes**	18.7 ± 1.3	29.3 ± 1.0 *	30.9 ± 1.5 *	26.6 ± 1.5 *	29.0 ± 1.6 *	21.0 ± 2.3
